# DeepGenMon: A Novel Framework for Monkeypox Classification Integrating Lightweight Attention-Based Deep Learning and a Genetic Algorithm

**DOI:** 10.3390/diagnostics15020130

**Published:** 2025-01-08

**Authors:** Abdulqader M. Almars

**Affiliations:** Department of Computer Science, College of Computer Science and Engineering, Taibah University, Yanbu 46421, Saudi Arabia; amars@taibahu.edu.sa

**Keywords:** monkeypox, deep learning, CNN, genetic algorithms, attention mechanism, pandemic

## Abstract

**Background**: The rapid global spread of the monkeypox virus has led to serious issues for public health professionals. According to related studies, monkeypox and other types of skin conditions can spread through direct contact with infected animals, humans, or contaminated items. This disease can cause fever, headaches, muscle aches, and enlarged lymph nodes, followed by a rash that develops into lesions. To facilitate the early detection of monkeypox, researchers have proposed several AI-based techniques for accurately classifying and identifying the condition. However, there is still room for improvement to accurately detect and classify monkeypox cases. Furthermore, the currently proposed pre-trained deep learning models can consume extensive resources to achieve accurate detection and classification of monkeypox. Hence, these models often need significant computational power and memory. **Methods**: This paper proposes a novel lightweight framework called DeepGenMonto accurately classify various types of skin diseases, such as chickenpox, melasma, monkeypox, and others. This suggested framework leverages an attention-based convolutional neural network (CNN) and a genetic algorithm (GA) to enhance detection accuracy while optimizing the hyperparameters of the proposed model. It first applies the attention mechanism to highlight and assign weights to specific regions of an image that are relevant to the model’s decision-making process. Next, the CNN is employed to process the visual input and extract hierarchical features for classifying the input data into multiple classes. Finally, the CNN’s hyperparameters are adjusted using a genetic algorithm to enhance the model’s robustness and classification accuracy. Compared to the state-of-the-art (SOTA) models, DeepGenMon features a lightweight design that requires significantly lower computational resources and is easier to train with few parameters. Its effective integration of a CNN and an attention mechanism with a GA further enhances its performance, making it particularly well suited for low-resource environments. DeepGenMon is evaluated on two public datasets. The first dataset comprises 847 images of diverse skin diseases, while the second dataset contains 659 images classified into several categories. **Results**: The proposed model demonstrates superior performance compared to SOTA models across key evaluation metrics. On dataset 1, it achieves a precision of 0.985, recall of 0.984, F-score of 0.985, and accuracy of 0.985. Similarly, on dataset 2, the model attains a precision of 0.981, recall of 0.982, F-score of 0.982, and accuracy of 0.982. Moreover, the findings demonstrate the model’s ability to achieve an inference time of 2.9764 s on dataset 1 and 2.1753 s on dataset 2. **Conclusions**: These results also show DeepGenMon’s effectiveness in accurately classifying different skin conditions, highlighting its potential as a reliable and low-resource tool in clinical settings.

## 1. Introduction

Monkeypox is a zoonotic illness caused by an infectious virus called the monkeypox virus. This virus belongs to the same group as the smallpox virus [1,2]. In 1970, the first individual case of this virus was documented in the Democratic Republic of the Congo [3]. Initially, this disease was identified in regions of Central and West Africa [4]. However, the virus began to spread globally to more than 50 nations, with over 3000 documented cases and one fatality [5]. Patients with the disease suffer from a rash, along with other symptoms such as fever, headache, muscle aches, and enlarged lymph nodes [6]. According to the World Health Organization (WHO), the monkeypox virus can be transmitted through direct contact with infected animals, individuals, or contaminated objects [7]. Public health organizations are extremely concerned about the rapid global spread of the monkeypox virus, especially given the lack of available cures for this disease. This situation highlights the urgent need for prophylactic measures to mitigate its growing effects [8].

In recent years, machine learning techniques and deep learning (DL) methods have become powerful tools for image analysis and pattern recognition, demonstrating significant potential for disease detection. These artificial intelligence (AI) methods can offer a rapid and promising solution for accurately identifying various skin diseases, including chickenpox, monkeypox, smallpox, measles, and others [9,10,11,12,13,14,15]. Furthermore, pre-trained models, such as EfficientNet, VGG19, MobileNet, and ResNet, have also been utilized for the detection of skin diseases, demonstrating their potential in this domain [5,11,16,17,18,19,20].

Explainable Artificial Intelligence (XAI) techniques have been utilized in various domains to identify important features and enhance the performance of the proposed models in the literature review [21,22,23]. Additionally, optimization techniques such as genetic algorithms, Bayesian optimization, and gradient search have been suggested to fine-tune deep learning models [24,25,26]. Although some of the proposed solutions can effectively classify skin diseases, their primary limitation lies in the high resource requirements, including substantial memory usage and GPU power, to achieve highly accurate results. In addition, to the best of our knowledge, this is the first study that integrates a lightweight convolutional neural network with an attention mechanism and a genetic algorithm. Another limitation is that most of the models are evaluated on a single dataset, which does not adequately reflect their generalizability or robustness across diverse real-world scenarios.

To address the limitations of existing work, this paper introduces a novel framework called DeepGenMon. The framework consists of three main components: an attention mechanism, a convolutional neural network (CNN), and a genetic algorithm (GA). First, the attention mechanism highlights and gives weights to particular areas of a picture that are crucial to the model’s decision-making process. This improves interpretability and performance by enabling DeepGenMon to concentrate on the most pertinent areas of the image. Second, the core of DeepGenMon is the CNN, which processes the visual input and extracts hierarchical features, enabling the classification of input data into multiple classes. Finally, the GA is used to fine-tune the hyperparameters of the CNN to improve the classification accuracy and robustness of the proposed model.

Optimization methods, such as grid search or Bayesian optimization, have been used recently in computer vision to enhance model performance by systematically exploring hyperparameter spaces and identifying the best configurations for improved accuracy [27,28,29]. However, these optimization methods are limited by predefined parameter sizes or have difficulty exploring complex search spaces. On the other hand, GAs use evolutionary strategies to effectively iteratively refine solutions, making them useful for optimizing hyperparameters in deep learning models, especially when computational resources are limited, because they can achieve near-optimal solutions in fewer iterations. In this study, the GA is selected because of its ability to efficiently explore a larger solution space and solve high-dimensional, non-convex optimization problems [30,31]. To showcase the effectiveness of DeepGenMon, comprehensive experiments were conducted on two publicly available datasets. The experimental results demonstrate that DeepGenMon can accurately identify various skin conditions.

The contributions of this paper can be summarized in four main points:

- A new model called DeepGenMon is suggested, which utilizes a lightweight, attention-based deep learning architecture to perform multi-class classification on different types of skin conditions.
- The suggested architecture incorporates an attention mechanism to enhance feature extraction and decision-making by concentrating on particular areas of the image that are most crucial for classification.
- The model’s hyperparameters are optimized using a genetic algorithm to achieve high performance.
- The model is tested on two publicly available datasets to illustrate their effectiveness. DeepGenMon achieves an overall accuracy of 98.5% on dataset 1 and 98.2% on dataset 2. The proposed approach shows an inference time of 2.9764 s on dataset 1 and 2.1753 s on dataset 2.

## 2. Related Works

Machine learning algorithms and deep learning techniques have shown outstanding results in computer vision tasks  [18,32,33]. Numerous studies have been proposed to address the challenge of skin disease classification using these advanced methods. For instance, an ensemble model named TMS, which integrates Transformer models with Support Vector Machines (SVMs), was suggested by Abdelrahim et al. for the classification of monkeypox lesions. When applied to classify monkeypox lesions into multiple classes, the suggested method achieved a classification accuracy of 95.45% [13]. Eid et al. [14] classified the monkeypox virus using a Meta-Heuristic Optimization of Long Short-Term Memory (LSTM). The suggested proposed model applied the Al-Biruni Earth Radius (BER) optimization algorithm to fine-tune the LSTM, improving its performance. Several statistical methods are employed to test the stability and performance of the proposed approach.

In 2023, Jaradat et al. utilized several pretrained deep learning models, including VGG19, VGG16, ResNet50, MobileNetV2, and EfficientNetB3, to classify monkeypox and identify the best-performing model [20]. The proposed model achieved an accuracy of 98.16%, a recall of 96%, a precision of 99%, and an F1-score of 98%. In another study, Ali et al. applied various pre-trained deep learning techniques, including VGG-16, ResNet50, and InceptionV3, to classify different types of diseases such as monkeypox, chickenpox, and measles. The models were evaluated and achieved an accuracy score of 82.96% [16].

In a recent study, an approach combining CNN models with a Beta-function-based normalization scheme was proposed for detecting monkeypox from skin lesion photos [34]. The model attains an average accuracy of 92.35%. Kumar leveraged several methods, including AlexNet, GoogleNet, and VGG16Net, along with machine learning algorithms such as SVM, KNN, Naïve Bayes, Decision Tree, and Random Forest, to identify skin diseases using the Monkeypox Skin Lesion Dataset  [35]. The highest accuracy score was achieved by VGG16Net, with an accuracy of 91.11%. Islam et al. [36] conducted an evaluation to assess the disease classification performance of seven advanced deep learning models using digital skin images. The models used in this study included MnasNet-A1, ResNet50, DenseNet121, Inception-V3, SqueezeNet, MobileNet-V2, and ShuffleNet-V2. The highest accuracy score was achieved by ShuffleNet-V2, with an accuracy of 79%.

In 2024, Maqsood et al. suggested a hybrid multi-stage fusion and selection framework for skin disease classification. The framework utilized several pre-trained deep learning approaches: Vision Transformers (ViTs), Shifted Window (Swin) Transformers, ResNet-50, ResNet-101, EfficientNetV2, and ConvNeXt-V2 [37]. In recent years, Khan et al. [9] applied the DenseNet-201 deep learning-based CNN model to analyze features and identify monkeypox conditions from skin photos, which were then used to train six different machine learning classifiers. This approach achieved an accuracy of 97.55%, outperforming the base DenseNet-201 model. Bala et al. [38] proposed a new modified model called MonkeyNet, which is based on DenseNet-201 and a CNN, for monkeypox classification. Additionally, the research incorporated the Grad-CAM tool to plot the model’s performance, identifying the regions of infection identified in each class image.

Furthermore, a lightweight convolutional neural network (CNN) was implemented for the classification of skin lesions across eight different diseases, achieving an accuracy score of 78% [39]. The Synthetic Minority Oversampling Technique (SMOTE) was also leveraged by Olusola et al. for the effective classification of melanoma skin diseases. The experimental results for a binary classification dataset showed a notable enhancement in melanoma detection, with an accuracy of 92.18% [40]. Sitaula and Shahi applied various pre-trained deep learning models (Xception, DenseNet-169, IncepResNetv2, and EfficientNet) for the classification of the monkeypox virus, achieving the highest accuracy rate of 87.13% [5]. Sharma et al. proposed a supervised learning-based classification method leveraging deep learning for the detection of monkeypox cases. The model was examined on a publicly available dataset from Kaggle and received an accuracy score of 96% [41]. Mehmood et al. presented a novel model, SBXception, which utilized the Xception network for classifying various skin lesions. The model was evaluated on the HAM10000 dataset, comprising 10,015 photos of skin conditions, and achieved an accuracy of 96.97% on the testing set [42]. Another study introduced a comprehensive analysis of U-Net and Transformer-based approaches for the identification of skin diseases. The study demonstrated that the hybrid approach reached an accuracy of 92.11% [43].

Table 1 summarizes the literature review, highlighting the main limitations and gaps. Although several studies have been conducted to address the challenges in skin disease classification, issues and limitations still persist, particularly in terms of generalization across diverse datasets and the complexity and intensive resource usage of these models. This underscores the need for further research and the introduction of lightweight models to address these challenges. To the best of our knowledge, this is the first study to integrate a lightweight convolutional neural network with an attention mechanism and a genetic algorithm for the classification of skin diseases.

## 3. Materials and Methods

**Figure 1:**
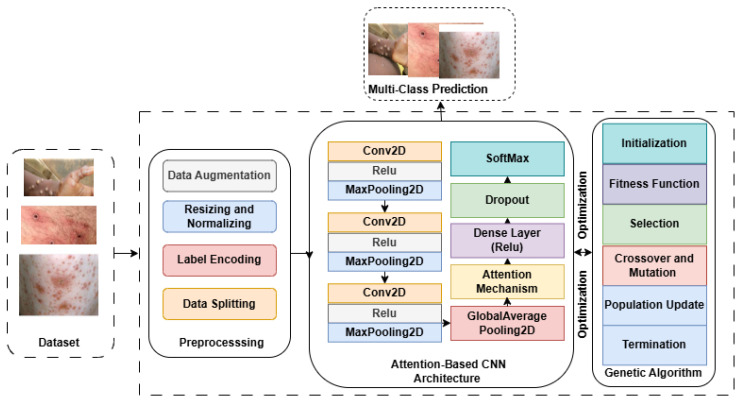
The proposed DeepGenMon architecture.

This study proposes a novel framework called DeepGenMon, which uses an attention-based CNN and a GA to analyze and detect skin conditions. DeepGenMon comprises five main steps: data collection and preprocessing, convolutional neural network (CNN) processing, attention mechanism, genetic algorithm, and classification. The key advantages of the proposed framework can be summarized as follows: (1) it is designed with fewer parameters and simpler architectures, making it less resource-intensive; (2) lightweight CNNs typically train faster and require less memory compared to deeper, more complex models, such as pre-trained ones; (3) it employs an attention mechanism to assign weights to specific regions of the image that contribute more significantly to the final decision; (4) it implments a GA to fine-tune hyperparameters and optimize the performance of the proposed model. The following section provides a detailed explanation of how these techniques were developed and evaluated. The architecture of DeepGenMon is illustrated in Figure 1.

### 3.1. Data Collection and Preprocessing

**Figure 2:**
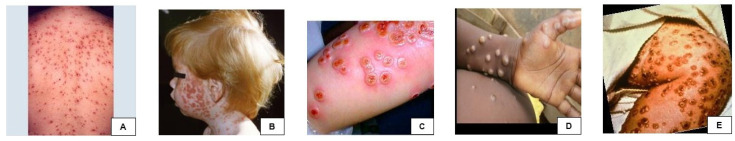
Samples from dataset 1 for each class, including (**A**) Chickenpox, (**B**) Measles, (**C**) Cowpox, (**D**) Monkeypox, and (**E**) Smallpox.

**Figure 3:**
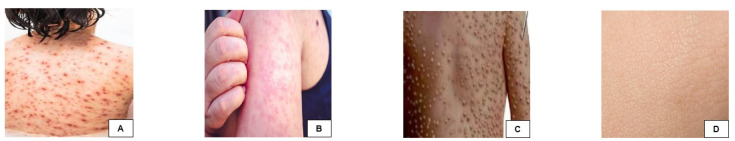
Samples from dataset 2 for each class, including (**A**) Chickenpox, (**B**) Measles, (**C**) Monkeypox, and (**D**) Normal.

In this study, two publicly available datasets were utilized for skin disease classification. The first dataset (https://www.kaggle.com/datasets/maxmelichov/monkeypox-2022-remastered/data?select=preprocessed_original_images (accessed on 10 March 2023)) contains a total of 847 files, categorized as follows: 178 chickenpox, 54 cowpox, 50 healthy, 47 measles, 160 monkeypox, and 358 smallpox files. Table 2 shows the distribution of images across different skin disease types in dataset 1 and dataset 2. Figure 2 displays examples from dataset 1. The second dataset (https://www.kaggle.com/datasets/sachinkumar413/monkeypox-images-dataset (accessed on 10 March 2023)) comprises 659 images, divided into 264 monkeypox, 100 chickenpox, 80 measles, and 215 normal images. Figure 3 displays examples from dataset 2. Following dataset collection for this stage, preprocessing was carried out to prepare the data for analysis. Preprocessing is a crucial step in improving model performance. The objective of this stage is to preprocess the data and transform them into a more meaningful representation for classification tasks. Furthermore, preprocessing techniques such as normalization, data augmentation, and image resizing are employed to reduce the impact of confounding factors such as skin tone, age, body part, pose, and lighting conditions. These steps help to standardize the images, reducing the impact of image variations. The preprocessing involves data augmentation, image resizing, normalization, label encoding, and data splitting. The specific preprocessing steps utilized in this study are as follows:

**1—Data Augmentation**: This study employed various data augmentation strategies to expand the size and diversity of the dataset. In the first dataset, the same augmented dataset provided in the repository was utilized. However, for dataset 2, approximately 15 augmented versions were generated for each image, providing a more comprehensive training set without the need for additional data collection. This helps to increase generalization, decrease overfitting, and improve model performance. The main modifications applied to the data are as follows:

- **Rotation:** Rotates the image within the specified range (±30 degrees).
- **Shifting:** Shifts the image vertically and horizontally by a fraction of the total height (0.2) and width (0.2).
- **Shearing:** Performs a shear transformation on the image (shear_range = 0.2).
- **Zooming:** Zooms in or out on the image (zoom_range = 0.2).
- **Flipping:** Flips the image vertically and horizontally.

**2—Image Resizing and Normalization**: Since DeepGenMon employs a CNN, it requires input images of a fixed size. Consequently, all images in dataset 1 were scaled to 128 × 128 pixels, while images in dataset 2 were scaled to 244 × 244 pixels to maintain consistency and ensure optimal model performance. The selected image dimensions were informed by experimental results from prior studies, which showed that a slightly smaller image size could improve classification accuracy for these types of specialized datasets [13,25,34].

After resizing, all images were normalized by scaling their pixel values to a range between 0 and 1. This was achieved by dividing each pixel value by 255, ensuring the data was prepared for optimal performance during the training of the DeepGenMon framework.

$${\mathrm{Nor\_Image}}_{i}=\frac{{\mathrm{Image}}_{i}}{255}$$

**3—Label Encoding**: The steps responsible for converting categorical labels (such as skin conditions: monkeypox, chickenpox, and healthy) to numerical values. We need to represent them as numbers for use in the proposed model.

**4—Data Splitting**: In this step, the dataset is split into training, validation, and testing sets using the following ratios: 70% of the total dataset is allocated to the training set, 15% for the validation set, and the remaining 15% for the testing set.

### 3.2. Convolutional Neural Natural (CNN)

A convolutional neural network (CNN) is a type of deep learning technique that is particularly successful for tasks such as image classification and has demonstrated exceptional performance across multiple domains [44,45]. This paper introduces a lightweight CNN to speed up training and inference while maintaining high performance and accuracy for analyzing and identifying skin conditions. By reducing computational requirements, it can be deployed on low-power devices with greater efficiency. The CNN consists of three main steps: convolution operation, activation function, and pooling. In the following, each step is explained in detail:

### 3.3. Convolution Operation

The convolutional layer is used to extract significant characteristics from the input data using kernels. The kernels slide across input images, extracting features at each location through mathematical operations. The convolution operation is calculated using the equation below:

(1) $$Cn\_O\left(i,j\right)=ReLU(\sum_{x=0}^{F-1}\sum_{y=0}^{F-1}\left(Inp_{i+x,j+y}\cdot K_{x,y}\right)+b)$$

where Cn_O(i,j) is the output feature map, $Inp_{i+x,j+y}$ is the value of the input image at position (i+x,j+y), $K_{x,y}$ represents the value of the kernal at position (i+x,j+y), and *b* is the bias term. The dimensions of the kernals is identified and optimized using a genetic algorithm.

### 3.4. ReLU Activation Function

As part of the convolution operation, the activation function relu (Rectified Linear Unit) is used element-by-element to the result to add non-linearity.

ReLU(x)=max(0,x)

### 3.5. Pooling

Then, max-pooling layers with a pooling window of size (2, 2) are applied to extract dominant features and reduce computation in subsequent layers. To calculate max-pooling, we use the following equation:

(2) $$\mathrm{PoolLayer}\left(i,j\right)=\max_{x,y}\left(Cn\_O_{i+x,j+y}\right)$$

The feature map is further reduced to a single vector by averaging the global averages using global average pooling.

### 3.6. Attention Mechanism

The attention mechanism is widely implemented across multiple fields, including natural language processing (NLP) and image analysis. [46]. The basic idea of this method is that it allows the model to give different weights to several parts of the input. The literature proposes several types of attention techniques, such as self-attention, multi-head attention, and global attention. Inspired by Bahdanau et al. [47], we implemented a simplified attention mechanism that enables our model to identify and place attention (weight) on the most relevant features within the input. The proposed approach is specifically designed to prioritize lesion-related features through the attention mechanism. This ensures that the classification process is driven by the morphological characteristics of the lesions rather than unrelated factors like body parts or lighting conditions. The following equations are used to calculate the average attention values for each input:

(3) $$\mathrm{Attention\_Scores}=\sum_{x=0}^{F-1}\sum_{y=0}^{F-1}\mathrm{input}\left(i+x,j+y\right)$$

Then, the attention scores are normalized and transformed into probabilities using the softmax algorithm.

(4) Attention_Weights=softmax(Attention_Scores)

(5) Weighted_Output=softmax(inputs∗Attention_Weights)

Furthermore, based on the weighted output, higher attention weights imply that those input components ought to have a greater impact on the final product. The outcomes of this phase are passed on to the following stage for classification.

### 3.7. Genetic Algorithm

The genetic algorithm (GA) is a meta-heuristic search method that belongs to the larger class of evolutionary algorithms and is motivated by the concepts of genetics and natural selection [24]. The core idea of the GA is to explore the solution space by leveraging historical data to guide the search toward regions with a higher potential for optimal performance. GAs are widely used for solving complex optimization and search problems, producing high-quality solutions by iteratively refining candidate solutions through operations like selection, crossover, and mutation.

In this paper, the GA has been selected due to its efficient exploration of a larger solution space and solve high-dimensional challenges and can achieve near-optimal solutions in fewer iterations [30,31]. In contrast to other optimization techniques, such as grid search or Bayesian optimization, which can be computationally costly, limited by predefined parameter intervals, or difficult to explore complex search spaces, GAs use evolutionary methods to effectively iteratively refine solutions, making them useful for optimizing hyperparameters in deep learning models, particularly when computational resources are constrained.

In this research, the CNN’s hyperparameters were optimized using a genetic method to improve its performance in classifying skin diseases. The learning rate, dropout rate, batch size, number of dense units, kernel size, and optimizer type are among the hyperparameters that have been adjusted. The hyperparameter space was investigated using the GA, producing optimal configurations through a series of steps (see Section 5).

- **1—Initialization:** This step creates a population of potential solutions (hyperparameter sets) at random. The size of the population is set to 10 in this study.
- **2—Fitness Function:** This stage computes the fitness scores of each individual in the population to evaluate the performance of a hyperparameter set. The higher the score, the better the individual’s fitness.
- **3—Selection:** Based on their fitness scores, this stage selects individuals for reproduction (crossover and mutation) and establishes which individuals will become parents.
- **4—Crossover:** Crossover techniques are applied here to generate new solutions (offspring) by combining features from selected parents.
- **5—Mutation:** The purpose of this step is to preserve diversity and investigate new regions of the solution space by introducing random mutations in the offspring. A small mutation rate is used to increase or decrease the parameter for numerical values.
- **6—Population Update:** In this step, we update the old population with the new offspring based on the crossover and mutation steps.
- **7—Termination:** The previous steps 2-6 are repeated for the designated number of generations or until a termination condition is satisfied.

By following these steps, the GA ensures a systematic search for CNN hyperparameters by searching optimal or nearly optimal solutions. The best hyperparameters generated by the GA are passed to the CNN to train and test the model.

### 3.8. Classification

In this step, a fully connected (dense) layer of 256 neurons is added to connect each neuron to the previous layer’s outputs. This layer also applied ReLU as an activation function. To prevent overfitting, we used dropout during the training process. A GA (see Section 3.7) identifies the optimal value for this hyperparameter, improving the model’s performance.

For multi-class classification, the output layer uses sparse categorical cross-entropy as the loss function. Furthermore, two types of optimizers (Adam and SGD) are tested using the GA to adjust the learning rate and improve convergence during training. The best hyperparameters are chosen based on the results of the GA. Finally, Softmax activation is used to convert the output logits into probabilities using the following equation:

(6) $$P\left(j\right)=\frac{\exp(z_{j})}{\sum_{k=1}^{C}\exp\left(l_{k}\right)}$$

where P(j) is probability of class *j*, $l_{i}$ is logit for class *i*, and *C* total number of classes.

## 4. Experiment and Evaluation

In this paper, comprehensive experiments were conducted on two publicly available datasets to evaluate the performance of DeepGenMon. The experimental details are explained in the following section, which discusses the evaluation metrics, baselines, and results obtained during the study.

### 4.1. Evaluation Metrics and SOTA

To test the performance of the DeepGenMon model, four well-known metrics are used: precision, recall, F1- score, and accuracy:

(7) $$Precision=\frac{True\;Postive}{True\;Postive+False\;Negative}$$

(8) $$Recall=\frac{True\;Postive}{True\;Postive+False\;Postive}$$

(9) $$F1-score=2\frac{precision★recall}{precision+recall}\;$$

(10) $$Accuracy=\frac{True\;Postive+True\;Negative}{True\;Postive+False\;Postive+False\;Negative+True\;Negative}$$

False positive (FP) denotes the number of samples that were mistakenly classified as positive, whereas true positive (TP) denotes the number of samples that were correctly classified as positive. Conversely, false negative (FN) represents the number of samples that were mistakenly classified as negative, while true negative (TN) indicates the number of samples that were correctly classified as negative.

Furthermore, to assess the performance of DeepGenMon, we use six popular models as the SOTA in our experimental comparisons: MobileNetV1 (lightweight), MobileNetV2 (lightweight), ResNet, EfficientNet, VGG16, Xception, and InceptionResNet. These models have been widely utilized in computer vision research and have proven their effectiveness in classification tasks [13,16,20,37].

The hardware requirements used in this study for implementing and evaluating the proposed model are a multi-core processor Intel i7 for general computation; a dedicated graphics card IRISX for accelerated training and inference of deep learning models; 16 GB of RAM; and a 256 GB SSD. The proposed lightweight DeepGenMon model comprises a total of 383,444 parameters, corresponding to approximately 1.46 MB. Among these, 127,814 parameters are trainable, accounting for about 499.27 KB, with no non-trainable parameters. Additionally, the optimizer requires 255,630 parameters, equating to approximately 998.56 KB.

## 5. Hyperparameter Study Using GA

In this section, we tested the influence of key hyperparameters on the performance of the proposed model using a GA. The hyperparameters under consideration include the learning rate (ranging from 0.0001 to 0.01), dropout rate (between 0.1 and 0.5), batch size (20, 40, or 60), number of dense units (64, 128, or 256), kernel size (between 3 and 7), and optimizer type (either Adam or SGD). The genetic parameters were set as follows: the number of generations is seven, the number of epochs is 50, the number of individuals in the population is 10, and the mutation rate = 0.1. The early stopping technique was also employed to monitor the training process and prevent overfitting. The optimal parameters and the corresponding results from applying the GA-tuned parameters before feeding them into the CNN are presented in Table 3 and Table 4. Based on the GA’s findings, the final set of best parameters was used to train the CNN model for classification, leading to an approximate accuracy rate of +1%. This demonstrates the significance of parameter optimization in enhancing model performance (see Table 5 and Table 6).

## 6. Results

The performance results of DeepGenMon, compared with state-of-the-art models, are presented in Table 5 and Table 6 (all scores are presented as percentages, with the best results highlighted in bold). Each model is assessed using four key metrics, as mentioned in Section 4.1 Based on the results from dataset 1 (Table 5), we provide the following insightful analyses. (1) MobileNetV1, MobileNetV2, and EfficientNet demonstrate very high performance across all metrics, indicating their effectiveness in identifying skin diseases. Compared with MobileNetV1, MobileNetV2 achieved a precision of 0.976, recall of 0.976, F1-score of 0.976, and accuracy of 0.976. Similarly, EfficientNet attained a precision of 0.977, recall of 0.977, F1-score of 0.977, and accuracy of 0.977. (2) Xception and InceptionResNet show a smaller performance improvement compared to the other models mentioned earlier. (3) ResNet’s and VGG16’s performance are significantly lower than the other models, with overall accuracies of 0.515 and 0.452, respectively. These results indicate that those models have significant difficulties in classifying several types of skin diseases.(4) DeepGenMon stands out with the highest scores across all metrics compared to the SOTA models, highlighting its ability to accurately identify different skin conditions.

**Figure 4:**
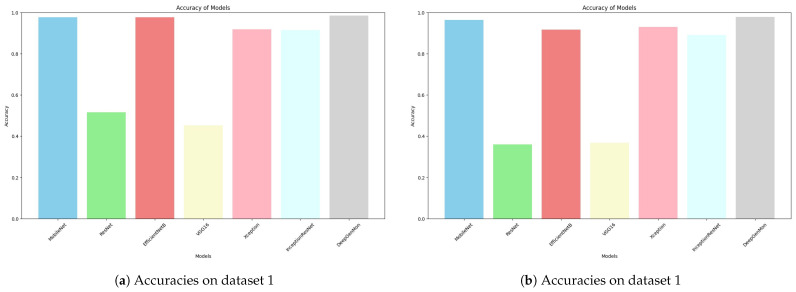
Comparative accuracies of DeepGenMon and state-of-the-art models.

In contrast, the results from dataset 2 (Table 6) reveal the following key analyses. (1) MobileNetV1 and MobileNetV2 continue to show reasonable performance, with only a slight decrease in metric values compared to dataset 1. (2) EfficientNet, Xception, and InceptionResNet also achieved adequate scores, with accuracies of 0.916, 0.929, and 0.891, respectively. (3) Similar to dataset 1, ResNet and VGG16 continue to exhibit the lowest performance among the models tested. (4) The proposed model, DeepGenMon, generally outperforms the other models in terms of precision (0.981), recall (0.982), F1-score (0.982), and accuracy (0.982). The comparative accuracies of DeepGenMon and state-of-the-art models are illustrated in Figure 4, showcasing the highest accuracy score of 0.985 in dataset 1 and 0.982 in dataset 2. Table 7 presents a comparison of the performance across various architectures, emphasizing the efficiency of DeepGenMon. It achieves the lowest inference times of 2.9764 s on dataset 1 and 2.1753 s on dataset 2.

**Figure 5:**
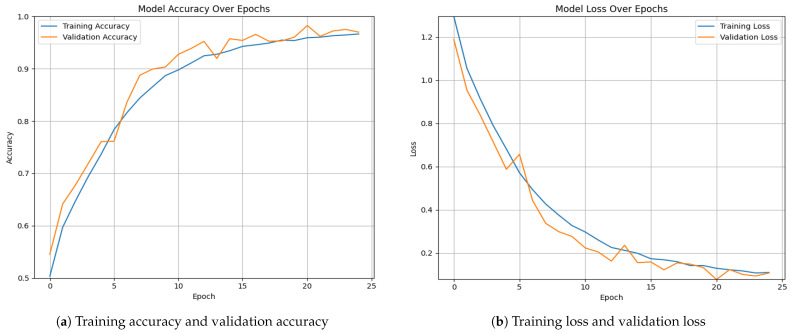
Overall accuracy and validation for the proposed framework on Dataset 1.

**Figure 6:**
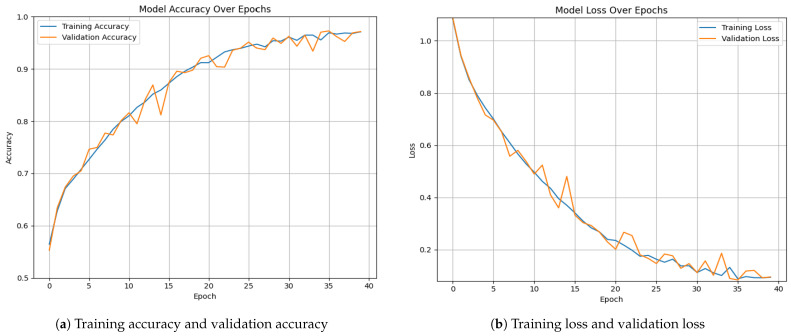
Overall Training and validation for the proposed framework on dataset 2.

Figure 5 and Figure 6 illustrate the overall accuracy and validation performance of the proposed model on the collected datasets with different epoch settings (the default value was set to 50, and early stopping was applied to prevent overfitting). On both datasets, it is evident that the model achieves high training and validation accuracy while maintaining low training and validation loss.

**Figure 7:**
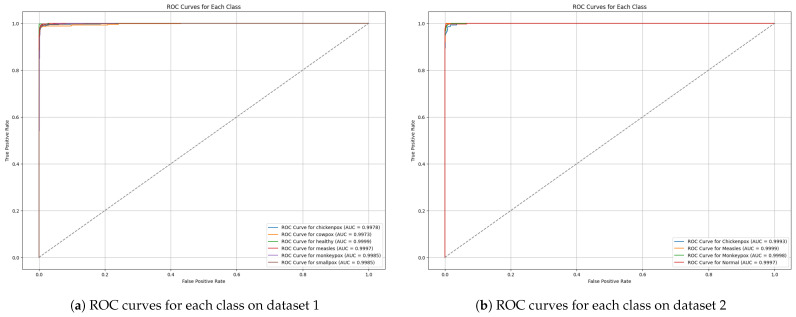
ROC curves for the proposed framework.

ROC curves are also utilized to assess DeepMonGen’s performance by displaying the trade-off between the true positive rate and the false positive rate. Figure 7 shows the ROC curves for the proposed framework on dataset 1 and dataset 2. As we can see from the figures, the results reveal that the ROC curve for all classes (e.g, chickenpox, monkeypox, measles, etc.) on both datasets is above 0.98, which indicates superior classification performance and a high degree of model capacity to discriminate between positive and negative classes.

**Figure 8:**
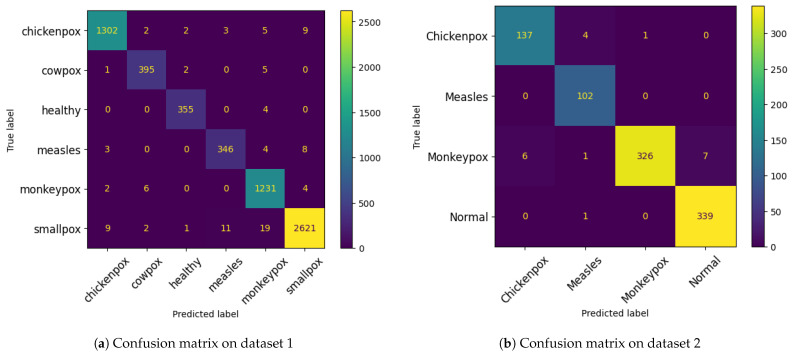
Confusion matrix for the proposed framework.

**Figure 9:**
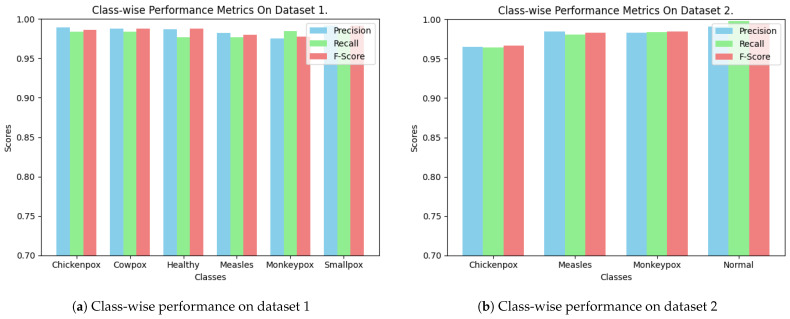
Class-wise performance of DeepGenMon.

Figure 8 displays the confusion matrix for the DeepGenMon model, indicating its classification test performance. The matrix provides a detailed breakdown of the instances of each class that the model correctly and incorrectly identified. According to the results, the model performs well across all classes and datasets, as measured by both confusion matrices. Nevertheless, there is a notable low error rate, indicating some classification challenges, particularly for specific conditions. The class-wise metrics for datasets 1 and 2 show that the DeepGenMon model performs exceptionally well across most classes, with no significant issues in recall, precision, or F-score (see Figure 9). This demonstrates the model’s ability to accurately distinguish between different conditions while maintaining consistent performance across all classes.

To sum up, the results clearly show that the DeepGenMon model outperforms all other SOTA models in terms of performance. This outstanding performance can be attributed to three key factors. First of all, the proposed model employed a variety of strategies, including early stopping techniques and regularization methods like dropout to prevent overfitting. These strategies ensure that the model does not recall the training data and instead learns generalizable features that may be applied to previously unseen data (test data). Second, the attention technique has significantly improved performance by allocating weights to the most relevant regions of the input images and focuses on key features required for accurate classification. Finally, the use of the GA as a hyperparameter optimization approach also contributes to the better performance of DeepGenMon by carefully exploring the hyperparameter space and identifying the optimal configuration.

## 7. Discussion

The suggested DeepGenMon outperforms the SOTA models with an overall accuracy of 98.5% on dataset 1 and 98.2% on dataset 2, proving its capacity to classify diverse skin diseases from images. Key advantages of DeepGenMon include its lightweight design, which significantly reduces computational resource requirements compared to existing models; faster training times; and consistently high accuracy. Its strong capacity to distinguish between various skin conditions makes it a reliable and cost-effective method in clinical settings, with the potential to improve diagnostic procedures. In other words, DeepGenMon’s lightweight architecture allows for seamless integration into existing systems without the need for substantial computational resources (e.g., hardware), making it an ideal solution for clinics. Furthermore, its efficiency can improve clinical operations by enabling faster decision-making, thereby enhancing the timely detection and treatment of skin diseases for patients.

Even though DeepGenMon produced encouraging results, it has three key limitations. First, it lacks transparency, which means it does not explain the model’s decisions. In real-world scenarios, clinicians need to understand how the model classifies different types of diseases and visualize the results effectively. Integrating Explainable AI (XAI) approaches such as SHAP and LIME can increase the model’s usability by offering interpretability and transparency for the model’s outcomes. Second, the datasets employed in this study are still relatively small, although the model was evaluated on two well-known datasets. Further evaluation on more diverse and larger datasets is required to confirm its robustness and applicability in broader scenarios. Finally, the model is optimized with the GA. Another direction for future research is to evaluate the model with advanced optimization techniques like the Sparrow algorithm and Particle Swarm Optimization.

## 8. Conclusions

This paper suggests a novel framework called DeepGenMon, which combines attention-based deep learning and a genetic algorithm to accurately identify various types of skin diseases, including chickenpox, melasma, monkeypox, smallpox, and others. The DeepGenMon applied an attention mechanism to focus on specific parts of an image that are critical to the model’s decision-making process. Following that, the CNN model is fine-tuned using a genetic algorithm to search for optimal hyperparameters and fine-tune values that enhance the detection of skin diseases. In this study, two publicly available datasets were used to evaluate the efficiency of the model. The results indicate that the model can achieve higher scores in terms of precision, recall, F-score, and accuracy. According to the experimental results, the proposed model outperforms state-of-the-art models, achieving an accuracy of 98.5% and 98.2%, respectively, on the collected datasets. In terms of inference time, the suggested model achieved a low inference time of 2.9764 s on dataset 2 and 2.1753 s on dataset 2. The proposed model offers significant advantages over the SOTA models by requiring fewer computational resources and fewer parameter sizes and enabling faster inference, making it well suited for real-time applications. Future work should involve testing the model on additional datasets from different regions to assess its generalizability and robustness across diverse contexts. Additionally, future research could explore the application of various interpretability techniques to better understand model decisions and enhance the interpretability and transparency of the model’s predictions.

## Figures and Tables

**Table 1:** Summary of papers on monkeypox detection and classification.

Citation	Method	Accuracy Score	Dataset	Limitation
Abdelrahim et al. [13]	Ensemble Model Transformer with SVM	95.45%	Monkeypox Skin Lesion Dataset	Evaluated on a single dataset, which may limit its generalizability to other datasets
Eid et al. [14]	LSTM with Meta-Heuristic Optimization	Not Mentioned	Monkeypox Dataset	Evaluated on a single dataset and uses only statistical methods to evaluate the model
Jaradat et al. [20]	GG19, VGG16, ResNet50, MobileNetV2, and EfficientNetB3	98.16%	Data monkeypox	Tested on a single dataset and relied on high-resource models
Ali et al. [16]	ResNet, VGG-16 and InceptionV3	82.96%	Monkeypox Skin Lesion Dataset	Tested on a single dataset and low accuracy
Pramanik et al. [34]	CNN models with a Beta function- based normalization scheme	92.35%	Monkeypox Skin Lesion Dataset	Evaluated on a single dataset and only applied binary classification
Kumar [35]	AlexNet, GoogleNet and VGG16Net, SVM, KNN, Naïve Bayes, Decision Tree and Random Forest	91.11%	Monkeypox Skin Lesion Dataset	Evaluated on a single dataset, which may limit its generalizability to other datasets
Maqsood et al. [37]	Vision Transformers (ViT), Shifted Window Transformers, ResNet-50, ResNet-101, EfficientNetV2, and ConvNeXt-V2	98.64%	Monkeypox Skin Lesion Dataset	Relied on high-resource and complex models
Hussain et al. [36]	MnasNet-A1, ResNet50, DenseNet121, Inception-V3, SqueezeNet, MobileNet-V2, and ShuffleNet-V2	79%	Monkeypox Skin Lesion Dataset	Low accuracy
Khan et al. [9]	DenseNet-201 deep learning-based CNN model	97.55%	MPox dataset	Evaluated on a single dataset and relied on high-resource and complex models
Bala et al. [38]	DenseNet-201 deep learning-based CNN model	98.91%	Monkeypox Skin Images Dataset	Evaluated on a single dataset and relied on high-resource and complex models
Sandeep et al. [39]	A low-complexity CNN	78%	Not mentioned	Low accuracy
Abayomi-Alli et al. [40]	SMOTE	92.18%	PH2 dataset	Evaluated on a single dataset, which may limit its generalizability to other datasets
Ahsan et al. [18]	Xception, DenseNet-169, IncepResNetv2 and Efficient	85.44%	Monkeypox2022	Low accuracy
Sharma et al. [41]	Supervised learning-based classification method	96%	Open-source dataset from Kaggle	Evaluated on a single dataset, which may limit its generalizability to other datasets
Mehmood et al. [42]	Xception Network	96.97%	HAM10000 Dataset	Evaluated on a single dataset, which may limit its generalizability to other datasets

**Table 2:** Distribution of images across different skin diseases.

Type	Chickenpox	Cowpox	Measles	Monkeypox	Smallpox	Healthy	Total
Dataset 1	176	54	47	160	358	50	847
Dataset 2	100	-	80	264	-	215	659

**Table 3:** All configurations and accuracies on dataset 1.

Learning Rate	Dropout Rate	Batch Size	Dense Units	Kernel Size	Optimizer	Accuracy
0.007889	0.342074	20	256	5	sgd	0.418766
0.002890	0.434574	60	64	3	adam	0.887909
0.002888	0.475651	20	64	3	adam	0.904754
0.004991	0.262347	20	128	3	adam	0.418766
0.008905	0.483051	40	256	7	sgd	0.973394
0.006093	0.373446	40	128	3	adam	0.601543
0.002614	0.365266	20	64	5	adam	0.857053
0.002525	0.361512	40	128	5	sgd	0.933249
0.009092	0.120170	60	256	3	adam	0.973161
0.008759	0.334553	40	64	5	adam	0.949622
0.002890	0.434574	60	64	3	adam	0.965995
0.007889	0.342074	20	256	5	sgd	0.966310
0.008397	0.412562	30	256	6	sgd	0.929471
0.002889	0.437656	40	64	3	adam	0.925220
0.002888	0.475651	20	64	3	adam	0.923961
0.004991	0.262347	20	128	3	adam	0.899087
0.008905	0.483051	40	256	7	sgd	0.418766
0.005390	0.388324	40	160	4	adam	0.418766
0.006440	0.326633	20	192	4	adam	0.952771
0.005389	0.408862	20	160	4	adam	0.945686
0.007889	0.342074	20	256	5	sgd	0.971902
0.002890	0.434574	60	64	3	adam	0.964106
0.002889	0.437656	40	64	3	adam	0.916247
0.002888	0.475651	20	64	3	adam	0.971115
0.008143	0.377318	25	256	5	sgd	0.942065
0.008397	0.412562	30	256	6	sgd	0.662941
0.000100	0.436115	50	64	3	adam	0.660422
0.005390	0.388324	40	160	4	sgd	0.696788
0.005644	0.423568	45	160	4	sgd	0.418766
0.096395	0.455112	40	64	3	adam	0.938917
0.007889	0.342074	20	256	5	sgd	0.985598
0.008016	0.359696	22	256	5	sgd	0.954660
0.002889	0.436115	50	64	3	adam	0.955290
0.002889	0.437656	40	64	3	adam	0.946946
0.002890	0.434574	60	64	3	adam	0.939389
0.002888	0.475651	20	64	3	adam	0.827613
0.008143	0.377318	25	256	5	sgd	0.418766
0.005389	0.408862	20	160	4	sgd	0.850756
0.005390	0.388324	40	160	4	adam	0.970560
0.005389	0.389865	30	160	4	adam	0.968042
0.008016	0.359696	22	256	5	sgd	0.962374
0.002890	0.434574	60	64	3	adam	0.980403
0.007889	0.342074	20	256	5	sgd	0.952928
0.002889	0.437656	40	64	3	adam	0.958753
0.002889	0.436115	50	64	3	adam	0.966152
0.005389	0.389094	35	160	4	adam	0.418766
0.005453	0.397905	36	160	4	sgd	0.845403
0.005389	0.389865	30	160	4	sgd	0.665145
0.005390	0.388324	40	160	4	adam	0.418766
0.000100	0.350885	20	256	6	sgd	0.693955

**Table 4:** All configurations and accuracies on dataset 2.

Learning Rate	Dropout Rate	Batch Size	Dense Units	Kernel Size	Optimizer	Accuracy
0.0016	0.3623	38	188	3	adam	0.7122
0.0016	0.2642	20	128	3	adam	0.6742
0.0087	0.4196	20	128	3	adam	0.7637
0.0091	0.2683	60	64	4	adam	0.9806
0.0070	0.4948	20	64	4	sgd	0.9835
0.0043	0.3798	20	128	4	sgd	0.8062
0.0048	0.2829	60	256	3	sgd	0.6169
0.0016	0.2082	40	64	3	sgd	0.5748
0.0019	0.3377	60	256	3	sgd	0.5868
0.0058	0.3899	40	128	3	adam	0.3786
0.0016	0.2689	60	256	3	adam	0.9744
0.0016	0.2666	40	192	3	adam	0.9823
0.0016	0.2642	20	128	3	adam	0.3786
0.0051	0.3442	40	192	3	adam	0.7604
0.0053	0.2663	40	96	3	adam	0.7167
0.0051	0.3419	20	128	3	adam	0.9806
0.0091	0.2683	60	64	3	adam	0.9394
0.0070	0.4948	20	64	4	sgd	0.9563
0.0043	0.3795	20	96	3	sgd	0.6429
0.0087	0.4196	20	128	3	adam	0.9621
0.0016	0.2677	50	224	3	adam	0.9761
0.0016	0.2642	20	128	3	adam	0.9790
0.0016	0.2666	40	192	3	adam	0.9819
0.0016	0.2666	40	192	3	adam	0.9509
0.0016	0.2654	30	160	3	adam	0.9431
0.0034	0.3066	50	224	3	adam	0.9819
0.0016	0.2689	60	256	3	adam	0.9794
0.0034	0.2887	50	176	3	adam	0.9765
0.0051	0.3442	40	192	3	adam	0.9728
0.0053	0.2663	40	96	3	adam	0.9769
0.0016	0.3485	45	208	3	adam	0.9765
0.0016	0.2654	30	160	3	adam	0.9786
0.0016	0.2659	35	176	3	adam	0.9765
0.0016	0.2654	30	160	3	adam	0.9786
0.0016	0.2666	40	192	3	adam	0.9798
0.0016	0.2642	20	128	3	adam	0.9794
0.0016	0.2677	50	224	3	adam	0.9806
0.0016	0.2666	40	192	3	adam	0.9761
0.0016	0.2671	45	208	3	adam	0.9720
0.0016	0.2666	40	192	3	adam	0.9790
0.0016	0.2654	30	160	3	adam	0.9794
0.0016	0.2659	35	176	3	adam	0.9794
0.0016	0.3075	42	200	3	adam	0.9823
0.0016	0.3072	39	192	3	adam	0.9835
0.0016	0.3485	45	208	3	adam	0.9794
0.0016	0.2666	40	192	3	adam	0.9757
0.0016	0.2654	30	160	3	adam	0.9814
0.0016	0.2657	32	168	3	adam	0.9715
0.0016	0.3069	37	184	3	adam	0.9814
0.0016	0.3069	37	184	3	adam	0.9773

**Table 5:** Model performance metrics compared with state-of-the-art models on dataset 1.

Model	Precision	Recall	F1-Score	Accuracy
MobileNetV1 (Lighweight)	0.955	0.946	0.955	0.945
MobileNetV2 (Lighweight)	0.976	0.976	0.976	0.976
ResNet	0.577	0.515	0.438	0.515
EfficientNet	0.977	0.977	0.977	0.977
VGG16	0.451	0.452	0.429	0.452
Xception	0.921	0.920	0.920	0.919
InceptionResNet	0.919	0.915	0.915	0.915
DeepGenMon	0.985	0.984	0.985	0.985

**Table 6:** Model performance metrics compared with state-of-the-art models on dataset 2.

Model	Precision	Recall	F1-Score	Accuracy
MobileNetV1 (Lighweight)	0.964	0.964	0.964	0.964
MobileNetV2 (Lighweight)	0.951	0.966	0.967	0.967
ResNet	0.241	0.36	0.201	0.36
EfficientNet	0.925	0.916	0.916	0.916
VGG16	0.368	0.368	0.368	0.368
Xception	0.935	0.929	0.929	0.929
InceptionResNet	0.906	0.891	0.892	0.891
DeepGenMon	0.981	0.982	0.982	0.982

**Table 7:** Inference times of various models on two different datasets.

Model	Inference Time (Dataset 1)	Inference Time (Dataset 2)
MobileNetV1	5.3227 s	6.6387 s
MobileNetV2	12.7219 s	8.5248 s
ResNet	14.3475 s	10.4155 s
EfficientNet	15.9458 s	13.8815 s
VGG16	5.1998 s	4.1703 s
Xception	10.0068 s	6.8989 s
InceptionResNet	26.8571 s	22.5141 s
DeepGenMon	2.9764 s	2.1753 s

## Data Availability

The data utilized in this study are publicly available: (1) https://www.kaggle.com/datasets/sachinkumar413/monkeypox-images-dataset (accessed on 10 March 2023). (2) https://www.kaggle.com/datasets/maxmelichov/monkeypox-2022-remastered (accessed on 10 March 2023).
